# Users’ Perception of Medical Blockchain

**DOI:** 10.34172/apb.2021.023

**Published:** 2020-07-07

**Authors:** Mehdi Dadkhah, Mohammad Mehraeen, Fariborz Rahimnia, Khalil Kimiafar

**Affiliations:** ^1^Department of Management, Faculty of Economics and Administrative Sciences, Ferdowsi University of Mashhad, Mashhad, Iran.; ^2^Department of Medical Records and Health Information Technology, School of Paramedical Sciences, Mashhad University of Medical Sciences, Mashhad, Iran.

## Dear Editor,

Nowadays, healthcare has been faced with different technologies that promise new advantages for better and more advanced healthcare. Blockchain is taken into consideration as one of the emerging technologies, which there are great interesting. According to Gartner, blockchain is classified as one of the top ten strategic technology trends in 2020.^1^ In current systems, a third-party control the transaction between requester and provider. For example, in the internet-based purchasing, the bank controls the financial transaction between customer and seller. In other words, there is a centralized control on the transactions, and consequently, the data is stored in the centralized database. Blockchain technology revolves the traditional transaction through decentralizing and centralized control center removal. All information about the completed transactions will be accessible for all participants in the network. This phenomenon leads to transparency in the transactions. Also, all participants are anonymous in the network, so it has more security. Blockchain was first introduced and created by Bitcoin, and it was utilized as the environment for cryptocurrency.^2^ According to *Forbes*, blockchain and 5G will lead to rethinking digital healthcare.^3^ In the pharmaceutical sector, blockchain can be used to ensure regulation and also preventing counterfeit drug distribution.^4,5^

Even there are research and developed systems for medical blockchain, the users’ perception of medical blockchain less discussed. In other words, we are not aware of users’/patients’ reactions to the blockchain. In this regard, we decided to analyze a social network to get insight. Social media analysis makes a deep and profound understanding of what people say and share about medical blockchain. This is also possible to detect sentiment behind their opinions. Social media are defined as the largest, most productive, and most dynamic sources for data dealing with human behavioral.^6^

For that purpose, we used Reddit (https://www.reddit.com) to gather data dealing with medical blockchain. Reddit comprises several benefits such as being a very active social community, providing public access to its content, and having a popular website.^7^ To gather contents related to the medical blockchain, the term “*title:medical blockchain*” has been searched in the Reddit. The *R* tool and library *“RedditExtractoR”* have been used to extract related content.^8,9^ About 195 posts plus 1300 comments dealing with the medical blockchain have been extracted (the extraction date was 13 January 2020). The library *“lexRankr”* has been employed to detect key sentences in the post.^10^ LexRank algorithm has been developed to calculate the relative importance of sentences in the document(s) and summarize texts.^11^ In this regard, the tutorials have been used to help for developing computer codes.^12,13^ The current study also analyzes comments in the term of sentiment. To analyze comments, the library *“SentimentAnalysis”* has been used.^14^ Sentiment analysis refers to the usage of computers for the detection of sentiments and emotions in the textual data.^15^

Based on the findings by using the mentioned method, the results are presented below. Based on published posts in Reddit, the introduction on blockchain sometimes refers to the usage of bitcoin infrastructure for medical data sharing in the posts. The usage of blockchain is interesting in electronic medical records (EMRs). It is due to users can check medical records in any place under safe and secure conditions. Then, treatment can be performed without geographical or time barriers. Also, the data will be up to date and integrated with any system. Users enumerated the main advantages of applying blockchain in healthcare, including improvement of security, privacy, integrity, as well as cost reduction, removing geographical limitations, providing a vast range of treatment selection, and holistic and connected data storage. The blockchain overcomes the weakness and barriers of the traditional medical system. In addition, the ownership of healthcare data will be returned to the patients. For many years, concerns about healthcare data security, privacy, and ownership were serious challenging matters. Whereas, the blockchain will solve and cover these troubles. Users stated a lack of cooperation between platform developers and medical data leaders as a significant obstacle, leading to limit acceptance and usage of the blockchain.

**Figure 1:**
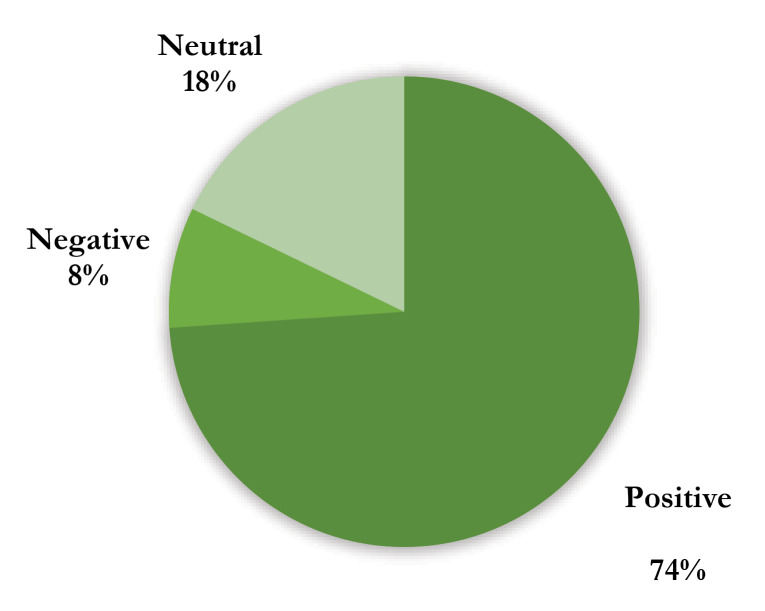
Percentage of sentiments in the comments.

Based on the published posts by users, they also discuss some developed medical blockchain systems such as FLETA’s eCRF, Medichain, Instant Access Medical ICO, Biohal, and Stem Cell Coin ICO. It seems that users are interested in these systems, and follow the development and growth of medical blockchain systems. It will be more interesting if we know the sentiment behind the comments, which provided by the users for the published posts in the Reddit. There are about 1300 comments for the mentioned 195 posts. Figure 1 shows the percentage of each sentiment concerning the comments. About 74 percent of the comments are positive, whereas only 8 percent of them are negative. This highlights the users’ positive enthusiasm in to medical blockchain. In the literature, Esmaeilzadeh and Mirzaei somewhat shared similar results. They concluded that patients have an attitude toward blockchain-based medical information exchange.^16^

Based on our analysis, it is expected that the research studying the tendency toward blockchain acceptance concludes the positive attitude of users toward blockchain. However, it should be considered that current users’ perception may be mainly associated with what they know and read about blockchain and not what they have experienced. Future research is also required to disclose the users’ perception of blockchain, considering the populations with previous usage experiences. Also, there is a requirement for research which discusses blockchain for medical applications, opportunities, challenges, etc. As EMR acceptance in some domains (such as aesthetic surgery practitioners) may be a challenging task,^17^ blockchain usage may be faced with such a barrier. In the pharmaceutical sector, there is a need for future research on the adoption of blockchain. Current research discloses the perception of users about medical blockchain, the perception of pharmaceutical professionals and stakeholders could be different. For future reading, please refer to cited references.^18,19^

The current letter is subject to about 195 posts and 1300 comments in the Reddit (top commented posts), not all the available published posts and comments in the Reddit. Also, it reflects Reddit users as the sample of people who may interact with medical blockchain (To see the distribution of users in Reddit refer to https://foundationinc.co/lab/reddit-statistics/). Additionally, Reddit users are usually more advanced in the term of technological knowledge from usual patients/blockchain users, and this may affect their perception. These would be considered as a limitation of the current study.

## Ethical Issues

Not applicable.

## Conflict of Interest

There is no conflict of interest.

## Acknowledgments

It is our pleasure to thanks Adam Spannbauer, data scientist in Thinkful, for assistance in R code checking and improvement.
